# Naturalistic climbing reveals adaptive strategies for interlimb coordination in freely moving mice

**DOI:** 10.1016/j.isci.2026.115901

**Published:** 2026-04-25

**Authors:** Christopher J. Black, Marco Beato, Liam E. Browne, Robert M. Brownstone, Stephanie C. Koch

**Affiliations:** 1 UCL Queen Square Institute of Neurology, University College London, London, UK; 2 Department of Neuroscience, Physiology, and Pharmacology, University College London, London, UK; 3 Wolfson Institute for Biomedical Research, University College London, London, UK

**Keywords:** Rodent behavior, Natural sciences, Kinematics, Biological sciences, Behavioral neuroscience, Biomechanics

## Abstract

Multi-limb coordination is vital for mammalian locomotion. While coordination requires reliable organization of limb movements, it also requires flexibility to adapt to environmental demands. In natural settings, animals must navigate diverse terrains that impose unique challenges. Climbing, for example, introduces constraints due to gravitational load, substrate variability, and the need for behaviors like reaching and grasping. Understanding how animals accommodate these demands provides insight into the flexibility of motor systems supporting locomotion. To address this, we developed a naturalistic climbing assay to investigate multi-limb coordination in freely moving mice. Climbing gait emulated aspects of horizontal locomotion, including a speed-dependent decrease in duty factor. However, coordination between homologous limb pairs was asymmetric, with forelimbs favoring anti-phase movements and hindlimbs showing a tendency toward in-phase movements. Notably, mice adjusted this strategy to overcome vertical gaps. Our results reveal that mice engage unique coordination strategies during climbing and adapt them to navigate vertical challenges.

## Highlights

• A naturalistic assay captures multi-limb coordination in freely climbing mice
• Climbing combines conserved gait features with distinct coordination strategies
• Forelimbs show anti-phase coordination; hindlimbs bias to in-phase coordination
• Mice flexibly reorganize interlimb coordination to overcome challenges

## Graphical abstract

**Figure:**
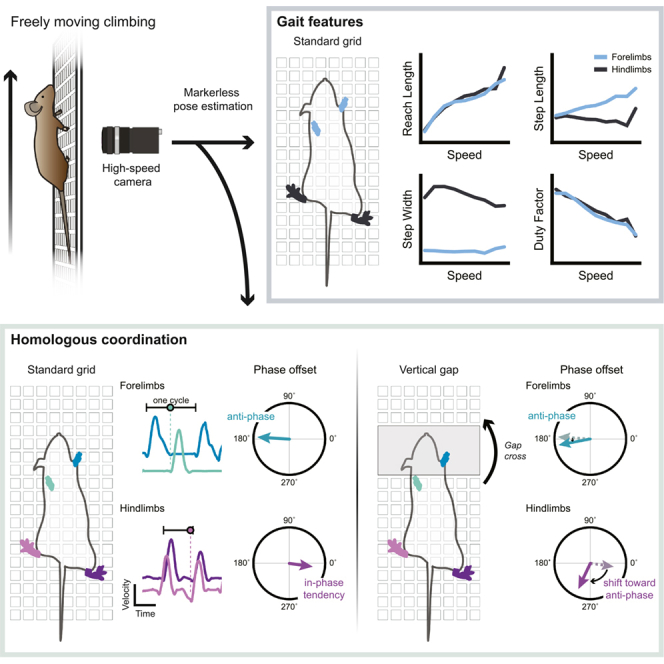

## Introduction

Locomotion is predicated on effective coordination across limbs, affording both efficient and adaptable movement.^1^^,^^2^^,^^3^ Effective coordination allows mammals to maintain rhythmic motion while flexibly adapting to changes in terrain, obstacles, or external perturbations. Across a range of species, horizontal locomotion has provided the dominant framework for understanding how coordination patterns adjust with speed and mechanical demand.^4^^,^^5^^,^^6^^,^^7^^,^^8^ These studies have defined how gait transitions, such as walking to trotting or galloping, emerge from systematic shifts in interlimb phase relationships and limb loading. However, when locomoting in the vertical plane, mammals must contend with gravity, balance, and substrate orientation in fundamentally different ways. Determining whether these changes impact coordination principles derived from horizontal movement is necessary for understanding the complex nature of adaptive motor control.

Climbing represents a common form of locomotion widely used by mammals for foraging, exploring, and escaping dangerous situations.^9^^,^^10^ Importantly, climbing challenges the principles established from horizontal gait analysis as the biomechanical strategies required to move against gravity in the vertical plane are fundamentally different. During climbing, animals must not only support body weight but also generate propulsive forces directed upward along a vertical substrate.^10^^,^^11^ Furthermore, climbing integrates quadrupedal locomotion, whole-body coordination, skilled multi-limb reaching and grasping, and postural control.^11^^,^^12^^,^^13^ These combined motor demands suggest that climbing may recruit distinct coordination strategies to maintain both effective limb contact patterns and flexibility under increased mechanical load.

Despite its ethological relevance, naturalistic climbing is challenging to examine quantitatively in mammals as limb movements are often partially or fully obscured in climbing environments making continuous tracking of complex movements difficult. As a result, existing work has primarily relied on coarse behavioral measures such as total distance or time spent climbing,^14^^,^^15^^,^^16^^,^^17^ providing limited insight into the fine-scale temporal relationships among limbs. The implementation of high-speed imaging to examine climbing mice in custom environments has enhanced quantitative analysis of limb kinematics, yielding biomechanical insights into limb use and gait dynamics.^18^^,^^19^ However, these approaches are still hindered by restricted limb viewing angles that limit our ability to investigate multi-limb coordination during vertical movement, which is a key component of adaptive motor control in complex environments.

To address this issue, we developed a novel climbing assay that leverages a modular transparent wall, high-speed ventral imaging, and markerless pose estimation to extract whole-body kinematics during naturalistic climbing in mice. We show that mice display an innate drive and ability to climb this novel vertical substrate without the need for training. We found that climbing kinematics preserves certain features of horizontal locomotion, most notably a speed-dependent decrease in duty factor, or the proportion of the stride spent in contact with the climbing surface. However, climbing also reveals distinct coordination patterns that deviate from canonical horizontal gaits. Notably, while forelimbs displayed highly consistent anti-phase movements, hindlimbs showed more variable patterns of coordination with a tendency toward in-phase movements. These results suggest an asymmetry in the roles and control strategies of forelimbs vs. hindlimbs during vertical movement, potentially reflecting the greater functional demands placed on the forelimbs for reaching, grasping, and postural support. Moreover, mice demonstrated the ability to flexibly adjust their coordination strategies to overcome vertical obstacles, underscoring the adaptive nature of climbing control.

Together, our findings suggest that while some core principles of horizontal locomotion are conserved across movement contexts, vertical locomotion engages unique interlimb coordination strategies. Studying naturalistic climbing offers a powerful window into how animals flexibly adapt locomotor patterns to meet biomechanical and environmental challenges. By enabling fine-grained quantification of limb coordination, our assay provides a foundation for uncovering neural and mechanical processes that support adaptive locomotion in three-dimensional environments.

## Results

### Untrained mice exhibit proficient climbing in a novel vertical locomotion assay

**Figure 1:**
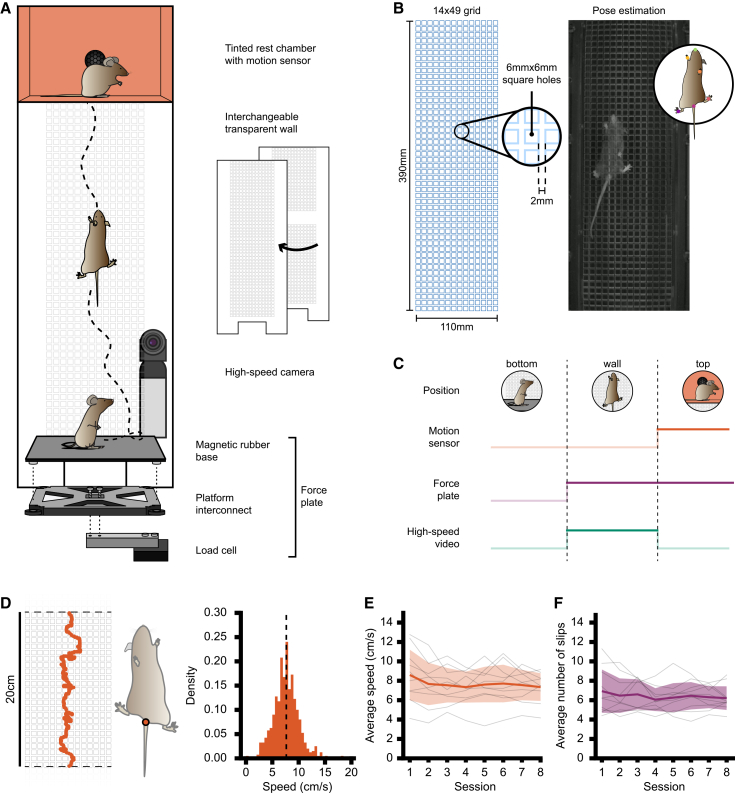
Vertical locomotion assay for kinematic analysis of climbing (A) Climbing apparatus consists of a custom force plate to track animals moving from the base to the wall, interchangeable transparent climbing wall, and resting chamber with motion sensor to detect successful climbs. A high-speed camera behind the climbing wall captures the ventral surface of the mouse during climbing. (B) Dimensions of the climbing grid layout (left) with an example frame extracted from a video (right) of a climbing mouse for use in markerless pose estimation. (C) Sensor-based video acquisition control scheme. (D) Trace of tail base (orange) for one climb within 20-cm region for calculating climbing speed (left). Histogram of speed (right) across all sessions was 7.7 ± 2.4 cm/s (n = 800 climbs, N = 10 mice). (E) Average speed across 8 testing sessions for all mice (mean ± SD, *n* = 10 mice, *p* = 0.98, Kruskal-Wallis). (F) Average number of slips per trial for each paw across 8 testing sessions for all mice (mean ± SD, *n* = 10 mice, *p* = 0.99, Kruskal-Wallis).

To examine multi-limb coordination during climbing, we designed a modular behavioral assay to perform high-speed imaging (200 frames per second) of the ventral surface of mice through a transparent climbing wall (Figures 1A and 1B). Social LEAP Estimates Animal Poses (SLEAP)^20^ was used to perform markerless pose estimation to track the nose, base of the tail, and paws during climbing (Figure 1B). High-speed video recordings were automated through Bonsai^21^ using a force plate at the base of the enclosure to detect climbing onset and trigger video acquisition and a motion sensor in the rest chamber to detect climbing offset and end video acquisition (Figure 1C).

Prior to behavioral testing, mice were habituated to the behavioral enclosure for a minimum of 3 days. During this time, 80% of mice spontaneously climbed (2.4 ± 2.0 climbs/session) to the top of the wall (Figure S1) as expected from previous home cage studies.^16^ To increase the number of climbs during testing, mice were gently coaxed toward the wall by lightly tapping their tail with a soft brush.

While mice displayed an innate ability to climb, we sought to determine if repeated climbing would improve their motor performance. To examine this, we looked at climbing speed and paw grasping accuracy across climbing sessions, both shown to be reliable markers of motor performance.^22^ Trial speed was calculated over a 20-cm area (Figure 1D) to account for differences in vertical starting positions between trials. We found no significant difference (*p* = 0.98) in average traversal speed across testing sessions (Figure 1E). Accuracy, as measured by the number of paw slips (Figure 1F), was also unchanged across sessions (*p* = 0.99). These results show that climbing speed and accuracy are consistent following repeated climbing.

### Climbing kinematics exhibits hallmarks of vertical locomotion

**Figure 2:**
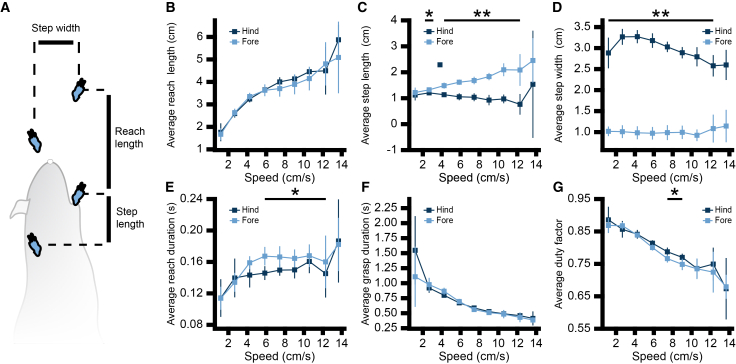
Climbing gait features alter with speed (A) Illustration of gait parameters extracted from pose estimation. Comparison of average forepaw (light blue) and hindpaw (dark blue) gait features of (B) reach length, (C) step length, (D) step width, (E) reach duration, (F) grasp duration, and (G) duty factor as a function of body speed in 1.6-cm/s bins (*n* = 10 mice). Points represent per-mouse mean ± 95% confidence interval. Statistical inference was performed using linear mixed-effects models (mouse as the experimental unit; ∗*p* < 0.05, ∗∗*p* < 0.001; see STAR Methods for bin inclusion criteria).

When locomoting on horizontal surfaces, gait is altered to adjust and maintain changes in speed.^4^^,^^23^^,^^24^^,^^25^ We hypothesized that climbing would follow similar principles. To test this, we first calculated correlation coefficients (Pearson’s r or Spearman’s ρ, as appropriate) between key gait features and climbing speed (Figures 2 and S2). There was a moderate positive correlation between speed and reach length (median coefficient [range] across mice; forepaw: 0.32 [0.24–0.47], hindpaw: 0.40 [0.37–0.56]), a negligible correlation between speed and step width (forepaw: −0.02 [−0.082 to 0.03], hindpaw: −0.19 [−0.37 to 0.00]), and a moderate negative correlation between speed and duty factor, defined as the ratio of the paw’s grasp duration to the total vertical stride duration (forepaw: −0.43 [−0.59 to −0.28], hindpaw: −0.41 [−0.69 to −0.15]), and speed and grasp phase (forepaw: −0.5 [−0.65 to −0.35], hindpaw: −0.38 [−0.70 to −0.31]) for fore- and hindpaws. Intuitively, these results show that as climbing speed increases, mice spend less time grasping. These results are also analogous to changes in gait during horizontal locomotion, where stance duration decreases as speed increases.^23^

During locomotion on sloped surfaces, gravitational forces shift weight toward the lower positioned limbs.^11^ We hypothesized that this redistribution of weight would lead to differences in gait parameters between fore- and hindlimbs, particularly across different speeds. To test this, we compared gait parameters between fore- and hindpaws as a function of speed (Figures 2B–2G) using linear mixed-effects models. We found that the average reach length did not differ between fore- and hindpaws (Figure 2B). Forepaws did have a significantly greater (*p* < 0.05) step length than hindpaws at speeds between 2 and 13 cm/s (Figure 2C), which reflects the difference in correlation between step length and speed (median [range] across mice; forepaw: 0.16 [0.08–0.27], hindpaw: −0.06 [−0.10 to 0.02]). Average step width was significantly greater (*p* < 0.001) for hindpaws than forepaws at all speeds (Figure 2D). The average reach durations were significantly greater (*p* < 0.05) for forepaws than hindpaws at speeds between 5 and 13 cm/s (Figure 2E), while the average grasp duration did not differ significantly between fore- and hindpaws at any speed (Figure 2F). Consequently, hindpaws exhibited a significantly higher (*p* < 0.05) duty factor than forepaws at speeds between 7 and 10 cm/s (Figure 2G). Together, these results suggest that the forelimbs help modulate climbing speed by taking larger steps to traverse more distance, whereas the hindlimbs may play a more supportive role by maintaining smaller steps with a wider base.

### Motor coordination is differentially regulated between fore- and hindlimbs during climbing

**Figure 3:**
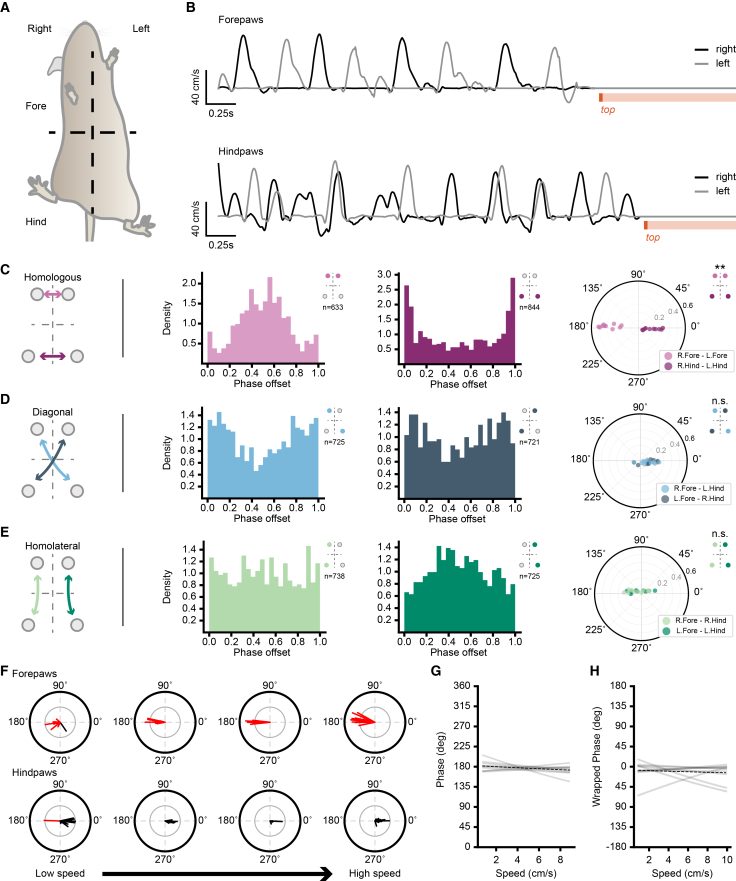
Climbing reveals distinct phase preference between homologous paw pairs (A) Reference body axis of climbing for ventral imaging. (B) Example velocity traces for forepaws (top) and hindpaws (bottom) for one climb illustrating motor coordination. (C–E) Coordination between homologous, diagonal, and homolateral paw pairs (light and dark shading specify relationship). (Left) Illustration of pair combination, (center left and center right) example histograms for corresponding paw pair, and (right) polar plots showing average phase preference in degrees and mean resultant vector for each animal (*n* = 10 mice, ∗∗*p* < 0.001, Watson-Williams). (F) Phase offset polar plots of homologous paw pairs in 2.05-cm/s bins between low (0.7 cm/s) and high (8.9 cm/s) speeds. Vector length reflects mean resultant vector (inner gray circle represents r = 0.4, outer black circle represents r = 0.8), and color indicates anti-phase (red) or in-phase (black) direction. (G and H) Individual (light gray) and mean (dotted black) circular-linear regression fits of phase offset and speed for (G) homologous forepaw pairs and (H) homologous hindpaw pairs (*n* = 10 mice).

As climbing requires both inter- and intrasegmental coordination, we next sought to examine how fore- and hindlimbs coordinate during vertical locomotion (Figures 3A and 3B). To quantify coordination, we calculated the phase offset between homologous, diagonal, and homolateral paw pairings across sessions (Figures 3C–3E).^1^ Homologous forepaw pairs (Figure 3C) exhibited an anti-phase preference (176.40 ± 3.50°, Rayleigh r = 0.99, *p* < 0.001), while homologous hindpaw pairs displayed an in-phase preference (352.19 ± 8.78°, Rayleigh r = 0.99, *p* < 0.001). Although the homologous pairs were significantly different from each other (Watson-Williams, *p* < 0.001), the clustering of phase offset values was broader for hindpaw pairs (Rayleigh r = 0.17 ± 0.09) compared to forepaw pairs (Rayleigh r = 0.39 ± 0.10). This suggests that while the forelimbs exhibit predominantly anti-phase coordination, hindlimb coordination is more variable, with a tendency toward in-phase movements.

Diagonal pairs (Figure 3D) displayed a significant in-phase relationship (right forepaw-left hindpaw: 342.53 ± 39.48°, Rayleigh r = 0.76, *p* < 0.05; left forepaw-right hindpaw: 342.97 ± 46.36°, Rayleigh r = 0.67, *p* < 0.05) with no significant differences between paw pairings (Watson-Williams, *p* = 0.98). Homolateral pairs (Figure 3E) exhibited a weak anti-phase relationship (left: 124.56 ± 61.90°; Rayleigh r = 0.41, *p* = 0.18; right: 128.99 ± 54.73°; Rayleigh r = 0.54, *p* = 0.048) with no significant difference between paw pairings (Watson-Williams, *p* = 0.90). However, across individuals, the distribution of phase offset values was weakly concentrated around the preferred phase for both diagonal (right forepaw=left hindpaw: 0.11 ± 0.07, left forepaw−right hindpaw: 0.13 ± 0.05) and homolateral (right: 0.11 ± 0.06, left: 0.11 ± 0.05) pairs. This reflects the variable coordination between fore- and hindlimbs. Together, these results suggest that climbing is defined by two cooperative motor strategies: robust alternating homologous forelimb coordination and adaptive, variable homologous hindlimb coordination.

Because phase offset assumes periodic limb movements and climbing is not strictly periodic, we next quantified inter- and intrasegmental coordination using a complementary, non-cyclic metric based on temporal overlap. To do this, we computed the Jaccard index for limb pairs with respect to overlap in reaching periods during climbing (Figure S3). This Jaccard index yields values between 0 and 1, where 0 indicates no temporal overlap, 0.5 indicates approximately 50% temporal overlap, and 1 indicates complete temporal overlap. Similar to the phase offset analysis, mean Jaccard values for homologous forelimb pairs (0.011 ± 0.005) were significantly lower (estimate = −0.149, standard error (SE) = 0.004, *p* < 0.001) than hindlimb pairs (0.160 ± 0.027). Importantly, although hindlimb pairs exhibited greater temporal overlap than forelimb pairs, Jaccard values remained well below 0.5, indicating that hindlimb reaching was not predominantly synchronous. Neither were diagonal paw pairs significantly different from each other (right forepaw−left hindpaw: 0.108 ± 0.044, left forepaw−right hindpaw: 0.113 ± 0.035, estimate = 0.006, SE = 0.004, *p* = 0.13) nor were homolateral pairs (right: 0.053 ± 0.016, left: 0.054 ± 0.024, estimate = 0.003, SE = 0.004, *p* = 0.47).

Coordination between homologous limb pairs is dependent on speed during horizontal locomotion.^3^^,^^4^ However, during vertical locomotion, maintaining contact to the climbing substrate is paramount, and therefore, certain coordination strategies may be preserved to avoid loss of contact. Therefore, we hypothesized that speed would have less influence on homologous coordination strategy during climbing. To examine this, we first sorted phase offset values for homologous paw pairs into four bins based on body speed (between 0.7 and 8.9 cm/s). We found that across these four bins, mice maintained predominantly anti-phase forelimb coordination and in-phase hindlimb coordination (Figure 3F). In the slowest bin, we noticed a greater variance in phase preference (Figure S4). Additionally, some instances of slow body speeds corresponded to periods of idleness producing increased lift-off durations for phase calculations that artificially generated in-phase values (Figure S3; STAR Methods). We, therefore, restricted analysis to include only phase offset values where the lift-off duration was within the 90^th^ percentile of the total group (Figure S4). Following this thresholding, we then performed circular-linear regression on phase offset values and body speed to determine if phase offset between homologous pairs displayed any dependency on speed. For homologous forepaw pairs (Figure 3G), the average intercept was strongly anti-phasic (182.33 ± 12.72°, Rayleigh r = 0.97, *p* < 0.001) and the average slope (−0.01 ± 0.02) was not significantly different from zero (*p* = 0.22). For homologous hindpaw pairs (Figure 3H), the average intercept was strongly in-phase (−6.12 ± 23.40°, Rayleigh r = 0.92, *p* < 0.001) and the average slope (−0.01 ± 0.04) was also not significantly different from zero (*p* = 0.64, *t* test). These results show that, in contrast to horizontal locomotion, motor coordination in vertical climbing is not affected by speed, with forelimbs maintaining anti-phase coordination and hindlimbs maintaining in-phase coordination.

### Mice modulate coordination to meet environmental demands

**Figure 4:**
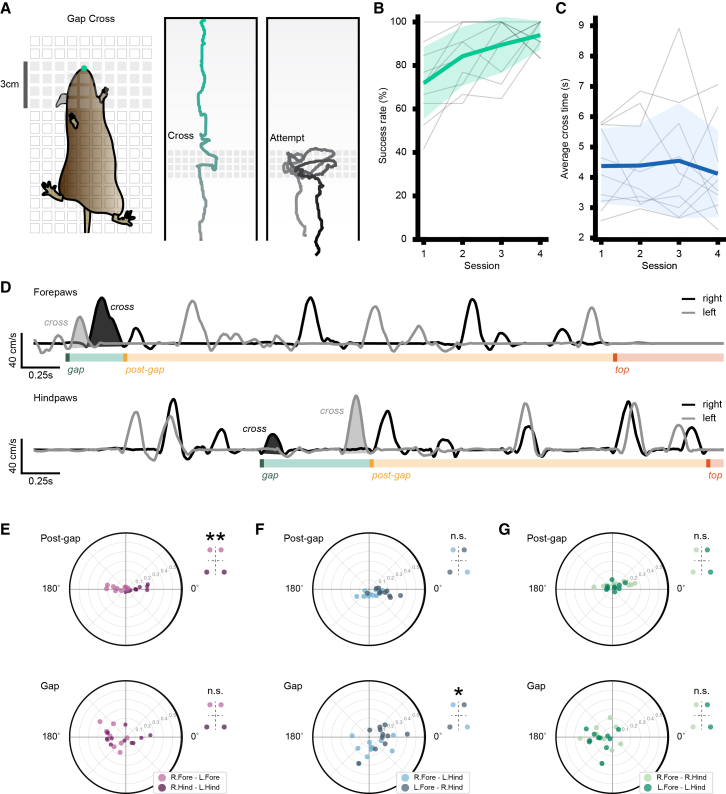
Coordination profile changes during gap cross task (A) Illustration of the modified gap cross wall (left) with example traces from the nose of successful (center) and attempted (right) crosses. (B) Gap cross success rate grand average (green, mean ± SD, *n* = 10 mice) and individual averages (gray) across 4 sessions. (C) Gap cross time-to-cross grand average (blue, mean ± SD, *n* = 10 mice) and individual averages (gray) across 4 sessions. (D) Example (top) forepaw and (bottom) hindpaw velocity traces during a single climb on the gap cross wall variant. (E–G) Polar plots for (E) homologous, (F) diagonal, and (G) homolateral paw pairs (light and dark shades specify limb groupings) show average phase preference in degrees and mean resultant vector for each animal (top) above the gap and (bottom) across the gap (*n* = 10 mice, ∗*p* < 0.05, ∗∗*p* < 0.001, Watson-Williams with Bonferroni correction).

In the wild, climbing surfaces are irregular and often unpredictable, imposing motor coordination challenges upon ascent. Having shown that mice have an optimal coordination strategy on the uniform climbing wall, we sought to determine whether this strategy would change when presented with a challenging vertical substrate. To test this, we designed a vertical version of the gap-cross task.^26^^,^^27^ We modified the standard grid of the uniform climbing wall by incorporating a vertical 3-cm (3.4 cm including the inter-grid spacing) section of solid etched acrylic spanning the width of the wall (Figure 4A). This formed a vertical tactile gap that mice had to cross to reach the top of the climbing wall. All mice were able to successfully cross the gap during the first session, and while the number of attempts decreased by the fourth day, there was no significant improvement in success rate or in average time to cross over the 4 sessions (Figures 4B and 4C). These results show that mice can innately overcome vertical obstacles.

We hypothesized that traversing the gap would require mice to alter their motor strategy and, therefore, coordination between limbs. To examine this, we next calculated the phase offset between paw pairs across the gap and after the gap to determine whether the phase preference of coordination for limb pairs changed. Above the gap, the phase offset of homologous forepaw (154.37 ± 68.45°) and hindpaw (350.98 ± 29.37°) pairs were significantly different (*p* < 0.001, Watson-Williams), exhibiting anti-phase and in-phase preferences, respectively (Figure 4E), similar to the standard grid. However, during gap crossing, the phase offset of homologous forepaw (190.82 ± 53.41°) and hindpaw (244.57 ± 105.98°) pairs was not significantly different (*p* = 0.81, Watson-Williams). Thus, the distinct phase preferences observed on the uniform portion of the grid converge during gap crossing such that the hindlimbs display a shift toward anti-phase coordination, indicating a reorganization of homologous coordination. This reorganization was also evident when coordination was quantified using the Jaccard index (Figure S5). Mean Jaccard values above the gap were significantly greater than mean Jaccard values across the gap for both homologous forepaw pairs (0.017 ± 0.008 vs. 0.004 ± 0.005) and hindpaw pairs (0.122 ± 0.042 vs. 0.054 ± 0.043). These differences were confirmed with linear mixed-effects models (forepaw: β = 0.013 ± 0.002, *p* < 0.001; hindpaw: β = 0.069 ± 0.013, *p* < 0.001). Although within-pair comparisons between above-gap and at-gap conditions were not statistically significant (forepaws: *p* = 0.72, hindpaws: *p* = 0.10, Watson-Williams), the loss of the homologous fore-hind distinction indicates a functional change in coordination strategy when traversing the gap.

When examining diagonal pairs (Figure 4F), the average phase offset (right forepaw−left hindpaw: 288.69 ± 58.74°; left forepaw−right hindpaw: 343.38 ± 30.23°) did not differ significantly (*p* = 0.07) and resembled coordination on the standard grid. While crossing the gap, diagonal pairs maintained a predominantly in-phase pattern (right forepaw−left hindpaw: 285.79 ± 52.99°; left forepaw−right hindpaw: 13.33 ± 37.70°) but became significantly different from each other (*p* < 0.05), suggesting an increase in asymmetry. However, within-pair comparisons between above-gap and at-gap conditions revealed no significant phase differences (right forepaw−left hindpaw: *p* = 1.00; left forepaw−right hindpaw: *p* = 0.23, Watson-Williams).

Finally, as in the standard grid, homolateral pairs above the gap (Figure 4G) displayed an in-phase preference (right forepaw−right hindpaw: 77.74 ± 69.00°; left forepaw−left hindpaw: 54.74 ± 80.00°; *p* = 1.00, Watson-Williams). When crossing the gap, both pairs shifted toward anti-phase coordination (right forepaw−right hindpaw: 173.74 ± 69.23°; left forepaw−left hindpaw: 184.09 ± 43.31°; *p* = 1.00, Watson-Williams). This shift was significant for both pairs when comparing the above-gap and at-gap phase offsets (*p* < 0.05, Watson-Williams), indicating that homolateral limb coordination also reorganizes during gap traversal. Together, these results demonstrate that coordination strategies are adaptable during climbing, with shifts in interlimb phase preferences reflecting the need to adjust to variable, irregular terrains.

## Discussion

Animals rely on different types of multi-limb coordination to successfully locomote across various landscapes and terrains. Here, we show that mice also employ different coordination strategies during vertical locomotion. Using a custom behavioral assay, we quantitatively characterized paw movements during wall climbing to show that forelimbs display robust anti-phase coordination, while hindlimbs exhibit more variable coordination with a tendency toward in-phase movements, irrespective of movement speed. Introducing a vertical gap in the climbing wall revealed that mice transiently reorganize their interlimb coordination to overcome obstacles, demonstrating flexible motor strategies adapted to changing environmental demands.

In some modes of horizontal quadrupedal locomotion, coordination between fore- and hindlimbs is mirrored.^28^ This was not the case during vertical climbing, where mice maintained alternating forelimb movements, while the hindlimb movements exhibited a tendency toward in-phase timing across a range of speeds. This coordination resembles the half-bounding gait characterized by sequential forelimb movement and synchronous hindlimb movement,^29^^,^^30^ which has been reported in arboreal species.^31^ Notably, however, half-bounding in horizontal locomotion occurs within a relatively narrow speed band, whereas during climbing this pattern persisted across multiple speeds. The apparent invariance of coordination across speeds likely reflects the functional demands of vertical locomotion, such as the need for greater contact on vertical vs. horizontal substrates, rather than dependence on movement speed alone. During climbing, maintaining wall contact with at least one forepaw is necessary to keep from falling. This in effect forces an anti-phase coordination between the upper limbs. Additionally, unlike walking or running, climbing relies on forelimb prehension, which likely contributes to the distinct interlimb coordination patterns observed. Coordination may also be affected by the specific dimensions and properties of the wall. For example, substrate diameter has been shown to influence gait dynamics in primates^32^^,^^33^ and harvest mice.^18^ Given the modularity of our custom climbing wall, future experiments can readily investigate the role of different wall features on climbing gait.

In nature, mice often climb irregular and varied terrains such as trees, shrubs, and grasses to forage for food.^34^^,^^35^^,^^36^ As these substrates are highly non-uniform, mice must adapt their climbing gait to navigate variations in substrate texture, size, and structure. This ability was apparent on the gap cross task, where mice altered their coordination strategy to successfully climb to the top of the wall. This flexibility is most likely tied to sensory feedback, which is critical to navigate irregular terrains, even in the vertical dimension.^37^ For example, tactile feedback is known to strongly modulate dexterous forelimb reaching and grasping,^38^ which is a major component of climbing. Whisking is also highly critical to sensorimotor integration in mice during locomotion. This is particularly true when dynamically avoiding obstacles at high speeds^39^ and when crossing gaps.^26^ Future studies will aim to leverage the modularity of our behavioral assay to investigate the contribution of different modalities of sensory feedback to coordination.

This work also opens the door to identify the neural circuits in interlimb coordination. In addition, such analysis may aid in the quantification of movement abnormalities in mouse models of disease. Climbing duration and distance have been used previously to assess motor deficits in conditions such as traumatic brain injury,^40^ pain,^15^ and neurodegenerative disease.^41^ Incorporating specific, detailed analysis of climbing kinematics could enhance the precision of motor assessments in these conditions. The recent advent of miniature microscopes for two-photon imaging has enabled the measure of calcium transients during wall climbing in mice.^42^ Using such tools in our naturalistic behavioral assay could reveal insights into the function of neural circuits involved in motor control and how such circuits support the precise multi-limb coordination necessary to achieve complex forms of locomotion.

### Limitations of the study

Despite these insights, several limitations should be considered when interpreting these findings. First, the adult mice (2-month postnatal) used in this study would likely have the prerequisite motor skills and prior practice climbing in their home cage, which is evidenced by spontaneous climbs during habituation and climbing consistency. This interpretation is further supported by developmental work showing that climbing-oriented harvest mice develop all the necessary biomechanical elements for climbing in the first postnatal month.^13^ Likewise, laboratory mice display climbing behavior within this same period.^43^ Therefore, behavioral testing during earlier developmental periods may reveal additional insight into the acquisition and refinement of skilled climbing.

Second, our analysis of locomotor speed was derived from movement of the tail base, which provides a global measure of body progression rather than a local measure of limb speed. Our metric, therefore, reflects a variety of behavioral components such as propulsion, pausing, and exploratory limb movements. Consequently, the speed bins analyzed may include multiple behavioral modes, which could obscure more subtle relationships between limb coordination and instantaneous locomotor dynamics. Future studies using multi-angle cameras with unsupervised pose-based behavioral segmentation approaches (e.g., Keypoint-MoSeq^44^) may allow for the identification of distinct behavioral phases during climbing, providing a more refined framework for linking interlimb coordination to specific movement strategies.

Finally, the present study focuses on kinematic measurements and does not quantify limb forces or mechanical stability during climbing. Previous work suggests that fore- and hindlimbs play distinct mechanical roles during vertical locomotion, with the upper limbs exerting tensile forces and the lower limbs exerting compressive forces.^11^ While such differences in limb-substrate interaction may provide a potential explanation for the distinct homologous coordination observed between fore- and hindlimbs, without direct measurements of substrate reaction forces, the mechanical contributions of individual limbs cannot be resolved. Integrating force measurements in future studies could help link coordination strategies to mechanical forces generated during climbing.

## Resource availability

### Lead contact

All requests for further information and resources should be made to and will be fulfilled by the lead contact, Stephanie C. Koch (s.koch@ucl.ac.uk).

### Materials availability

This study did not generate new unique materials.

### Data and code availability

• Markerless pose estimation data can be accessed from the following OSF repository and is publicly available as of publication (https://doi.org/10.17605/OSF.IO/JAZBK). All data reported in this paper will be shared by the lead contact upon request.
• Code related to the behavioral assay and data analysis has been deposited in an OSF repository (https://doi.org/10.17605/OSF.IO/JAZBK) and is also available on GitHub (https://github.com/cjblack/TheWall).
• Any additional information required to reanalyze the data reported in this paper is available from the lead contact upon request.

## Acknowledgments

The authors thank Dr. Laura Andreoli and Antonia Constantinescu from University College London for discussions on behavioral design and analysis. This work was supported by a Brain Research United Kingdom fellowship (to C.J.B.) and a 10.13039/501100009187Medical Research Foundation Fellowship MRF-087-0003-F-KOCH-C0917 (to S.C.K.).

## Author contributions

Conceptualization, data curation, formal analysis, investigation, methodology, software, validation, visualization, and writing – original draft, C.J.B.; project administration, resources, and writing – reviewing and editing, C.J.B., M.B., L.E.B., R.M.B., and S.C.K.

## Declaration of interests

The authors declare no competing interests.

## STAR★Methods

### Key resources table

**Table:** 

REAGENT or RESOURCE	SOURCE	IDENTIFIER
**Deposited data**		

Markerless pose estimation data from individual animals (OSF: JAZBK)	This paper	https://doi.org/10.17605/OSF.IO/JAZBK

**Experimental models: organisms/strains**		

Adult male mice	Charles River	C57/BL6J

**Software and algorithms**		

Python	Python Software Foundation	https://www.python.org/
Custom Python scripts	This paper	https://doi.org/10.17605/OSF.IO/JAZB
R	R core team	r-project.org
Bonsai	Bonsai	https://bonsai-rx.org/

**Other**		

Design files for climbing behavior	Dr. Christopher Black, UCL	https://doi.org/10.17605/OSF.IO/JAZBK and https://github.com/cjblack/TheWall

### Experimental model and study participant details

#### Animals

All experiments received review committee approval and were carried out at University College London on project license number 8884544. Procedures conformed to UK Home Office regulations and were performed in accordance with the UK Animals (Scientific Procedures) Act 1986. This study used equal numbers of adult (2-month-old) male and female C57/BL6J mice (sourced from Charles River). Mice used in this study were group housed in a 12-h light/dark cycle, in a temperature and humidity-controlled environment with *ad libitum* access to food and water.

### Method details

#### Climbing assay

Mice were habituated for 15–30 min in a small, padded chamber for 3–5 days with access to the climbing wall to gauge their propensity for spontaneous climbing. Mice were placed at the bottom of the climbing enclosure and were allowed to explore. When mice climbed to the top chamber, they were placed back to the bottom of the enclosure within 2 min. During testing, mice either climbed spontaneously or, if spontaneous climbing did not occur within 1 min, were gently coaxed with light taps from a paint brush to initiate climbing. This ensured repeated trials across sessions and reduced overall time mice spent performing behavior. Mice completed single sessions (<30 min) consisting of no less than 10 climbs per session. Mice underwent 4–5 sessions per week and completed a minimum of 8 sessions for the standard wall.

Following the completion of behavioral testing on the standard wall, mice completed four additional sessions on the gap cross wall. For each session mice were manually coaxed to initiate wall climbing until 10 successful gap crosses were made, with no more than 25 attempts allowed during any given session. Trials where mice did not cross the gap but their nose and or forepaws did, were considered attempted crossings.

#### Behavioral rig

The main compartment of the climbing rig was a custom laser cut enclosure supported by 25 mm rails on an aluminum breadboard (Thorlabs). A custom matte-black acrylic door and detachable magnetic wall (Perspex, Cutlaser Cut LTD) were used to easily place animals in the enclosure and clean the behavioral rig. 3 mm transparent acrylic side walls (Perspex, Cutlaser cut LTD) were used to observe the animal during experimental sessions.

The base chamber of the climbing rig consisted of a custom 3D printed (Protolabs Network, 3D Hubs) compartment housing a removable magnetic base plate attached to a 1 kg load cell (Adafruit, 4540). Output from the load cell was sent to an analog-to-digital converter (HX711, SparkFun), before being read-in and thresholded by an Arduino microcontroller to identify climbing initiations.

The climbing wall consisted of a single 2 mm thick acrylic sheet with a custom grid that was laser cut to a final size of 400 × 225mm (Perspex, Cutlaser Cut LTD). The standard grid wall was a 14 × 49 arrangement of 6 mm square holes evenly spaced by 2 mm, while the gap variant was created by etching, as opposed to cutting, a 14 × 4 section of the grid to produce a tactile surface that the mice could not grip. Illustrator files used for laser cutting are available at https://github.com/cjblack/TheWall.

A high-speed machine vision camera (1.6 MP Blackfly S, FLIR) with a wide-angle lens (4 mm UC Series Lens, Edmund Optics) was placed ∼37.5 cm behind the wall and rotated 90° for ventral imaging. Red (625 nm) LEDs were positioned behind the camera angled ∼45° to evenly illuminate the climbing wall. This provided optimal lighting for pose estimation while reducing unnecessary strain on the animal’s vision due to their decreased sensitivity past the 600 nm range.

The top chamber consisted of a custom 3 mm thick transparent red acrylic container (Perspex) designed to minimize light entry. At the back of the chamber was a motion sensor that sent a digital trigger to an Arduino whenever the animal entered the chamber.

Digital triggers tracking the load cell state and motion sensor were sent to a second Arduino microcontroller that communicated with the Bonsai-Rx software to automate high-speed video acquisition.

#### Markerless pose estimation

High-speed video data was collected at 200 fps and cropped to 600 × 1440 pixels. Prior to analysis, video data was corrected for rotational and wide-angle lens distortions. SLEAP was used to perform labeling, training, and inference for markerless pose estimation. 300 frames taken from 15 videos (20 frames/video) were labeled for the right and left fore- and hindpaws, base of the tail, and the nose. Labeled frames were then used to train a model with a UNet backbone using hyper parameters listed in Table S1. For the final model the mean error distance was 3.1 pixels (0.7 mm), the mean Average Precision (mAP) was 0.88, and the mean Average Recall (mAR) was 0.90. Training and inference were performed on a high-performance computing cluster with either an NVIDIA A100, Tesla V100, or Tesla P100 GPU.

#### Gait analysis

Speed for vertical gait analysis was calculated as the total distance traversed by the base of the tail during a given period, Δt. The base of the tail was selected as it most closely reflected movement of the torso and was not affected by individual movement of fore or hind limbs. Reaching and grasping was defined on every trial for each paw by first calculating the paw’s X and Y speed. Speed was then thresholded to find reach initiation events calculated as the time prior to when paw speed transitioned above threshold, and grasp events calculated as the time after paw speed transitioned below threshold. These time stamps were used for subsequent analyses for both gait and coordination. Paw slipping was determined by thresholding the paw speed to identify periods where the speed reached below −10 cm/s. This value was selected from identifying slipping events in videos to corresponding kinematic data.

Step length was calculated as the vertical (Y) distance between homologous paw pairs at the time of a grasping event, stride was calculated as the vertical distance between two consecutive grasps for one paw, and step width (base of support) was calculated as the horizontal (X) distance between homologous paw pairs. Reach phases were calculated as the time between reach initiation and subsequent grasp, while grasp phases were conversely calculated as the time between grasp and subsequent reach initiation. Duty factor was calculated as the ratio of time spent in grasp over the entire time of the climbing ‘stride’, which was defined as the time between subsequent grasping events. Gait parameters for forepaws and hindpaws were averaged into 1.6 cm/s bins based on body speed, which was calculated using the base of the tail.

#### Coordination analysis

To examine coordination, we used standard definitions for gait assessment. The climbing gait was determined by the time window in which each limb completed one ‘stride’. Strides for individual paws were determined by the period of successive wall contacts by the paw, which was determined by observing the rate of change in the X and Y coordinates. The phase offset was then calculated between paw pairings. Phase offset between a reference paw (P2) and a target paw (P1) is defined by adopting previous methodologies for calculating phase offset^45^:

$$\phi^{P2-P1}=\frac{\left(t_{s}-t_{0}\right)}{\left(t_{1}-t_{0}\right)}$$

Where [t_0_, t_1_] gives the period between consecutive reach initiations (lift-off duration) of a reference paw P2, and t_s_ is the initiation reach time of a target paw P1 during the reference period. Phase offsets were computed in both directions for each paw pairing, such that each paw served as the reference in turn, to avoid bias associated with selecting a single reference limb. Phase offset values were averaged for statistical consideration.

To compare phase offset by speed, we binned phase offset values for the different paw pairs in 2.05 cm/s bins based on body speed, which was again calculated using the base of the tail. Circular-linear regression of phase offset and body speed was performed using the *pycircstat2* python package.

In addition to phase offset, interlimb coordination was quantified using a temporal overlap metric based on the Jaccard index. For a given paw pair (P1, P2), the Jaccard index was defined as:

$$J\left(P2,P1\right)=\frac{\left|P1\cap P2\right|}{\left|P1\cup P2\right|}$$

Where P1∩P2 denotes the total duration where both paws were simultaneously engaged in reaching, and P1∪P2 denotes the total duration during which either paw was engaged in reaching. Jaccard values range from 0 to 1, with values near 0 indicating predominantly sequential reaching and values near 1 indicating highly synchronous reaching. Because this metric is based on absolute overlap in reaching time, it does not assume periodic gait cycles and is well suited to non-cyclic behaviors such as gap traversal.

### Quantification and statistical analysis

Data were hierarchically structured, with multiple strides nested within sessions and mice. For all statistical analyses, the mouse was treated as the experimental unit. To avoid pseudoreplication, measurements were averaged within mouse across sessions or across stride-level observations, as appropriate for each analysis.

For speed and slip characterization (Figure 1), each mouse contributed 80 climbs across 8 sessions (10 climbs per session). Normality was determined by the Shapiro-Wilks test. When data were normally distributed, parametric assumptions were satisfied; otherwise, non-parametric tests were applied. Non-parametric comparisons across groups were performed with the Kruskal-Wallis test. Resulting significance was determined with post-hoc tests between groups with Wilcoxon signed-rank test for paired data and Mann Whitney U test for un-paired data.

For all remaining analyses on the standard wall, each mouse contributed between 80 and 101 climbs, with a minimum of 10 climbs per session. Across mice for the standard wall, stride counts per paw ranged from 317 to 599 for right forepaws (median 364.5), 324–575 for left forepaws (median 384.5), 395–656 for right hindpaws (median 465.5), and 398–693 for left hindpaws (median 445.5). For the gap cross wall, each mouse contributed between 40 and 61 attempted climbs, of which 40–41 successful climbs were used for analysis. Across mice for the gap cross wall, stride counts per paw ranged from 153 to 296 for right forepaws (median 191.0), 180–287 for left forepaws (median 213.0), 203–305 for right hindpaws (median 248.5), and 198–292 for left hindpaws (median 239.0). Variation in stride counts across mice reflected differences in starting position and reach extent during climbing.

For analyses involving speed-binned comparisons of gait parameters (Figure 2), stride-level data were organised by bin and averaged within mouse, yielding one value per mouse per bin for each limb class (forelimb or hindlimb), with left and right limbs pooled within each class. Statistical inference was performed in R using linear mixed-effects models including speed, limb, and their interaction, with mouse included as a random intercept. Statistical inference was restricted to speed bins in which data from at least eight mice were available per limb. Differences between limbs at each speed bin were evaluated using pairwise contrasts of estimated marginal means derived from the fitted models. All speed bins are shown (Figure 2) for completeness, but bins with fewer contributing mice were considered descriptive. When comparing the relationship between gait features to speed (Figure S2), the Pearson correlation coefficient was calculated for reach length, step length, step width, reach duration, and duty factor. The Spearman correlation coefficient was calculated for grasp duration given its nonlinear relationship with speed.

For circular data (i.e., phase offset values for coordination analysis), measurements were pooled across sessions within each mouse and summarised as a single circular mean direction and resultant vector length using the *pycircstat2* Python package. Rayleigh’s r-test was then used to determine if circular distributions of mouse-level data were non-uniform (*p* < 0.05) and to identify the mean resultant vector, which indicates how disperse the data are with respect to the mean. Watson-Williams test was used to determine if there were significant differences across multiple groups of phase offset values for different paw pairings.

To provide a complementary, non-circular summary of interlimb coordination, temporal overlap between limb pairs was quantified using the Jaccard index. For visualisation, measurements were pooled across sessions within each mouse and averaged at the mouse level. Statistical inference was performed in R on the full observation-level dataset using linear mixed-effects models with mouse included as a random intercept to account for repeated measurements within animals. For the standard wall, limb pair was included as a fixed effect. For the gap cross wall, location (gap vs. post-gap) was included as a fixed effect, and models were fitted separately for homologous forelimb and hindlimb pairs. When models contained a single binary predictor, effects are reported as the fixed-effect coefficient (β). When multiple conditions or specific contrasts were evaluated, pairwise differences were calculated using estimated marginal means.

In all figures and text, the following convention was used: ∗*p* < 0.05, ∗∗*p* < 0.001, and n.s. for non-significant. Statistical tests and sample sizes are indicated in the figure legends and corresponding main text. Where full statistical reporting is not included in the legends and/or main text, additional details are provided in Table S2. Unless otherwise stated, values are reported as mean ± standard deviation across mice for linear variables and as circular mean ± circular standard deviation for circular variables. When appropriate, multiple comparisons were tested for using the Bonferroni correction to adjust *p*-values for significance.

## Supplemental information

Document S1. Figures S1–S5 and Table S1

Table S2. Detailed statistical results including tests, comparisons, test statistics, degrees of freedom, exact *p* value, and sample size (*n*) (A) Figure 2, (B) Figure 3, (C) Figure 4, (D) Figure S2, (E) Figure S3, (F) Figure S5
