# Supplementary material for: Naturalistic climbing reveals adaptive strategies for interlimb coordination in freely moving mice
Source: iScience. 2026 Apr 25;29(6):115901. doi: 10.1016/j.isci.2026.115901 (PMC13194540; doi:10.1016/j.isci.2026.115901)
Supplement: Document S1. Figures S1–S5 and Table S1 [file mmc1.pdf]

iScience, Volume *29*

Supplemental information

Naturalistic climbing reveals adaptive strategies for interlimb coordination in freely moving mice

Christopher J. Black, Marco Beato, Liam E. Browne, Robert M. Brownstone, and Stephanie C. Koch

Supplemental Figures

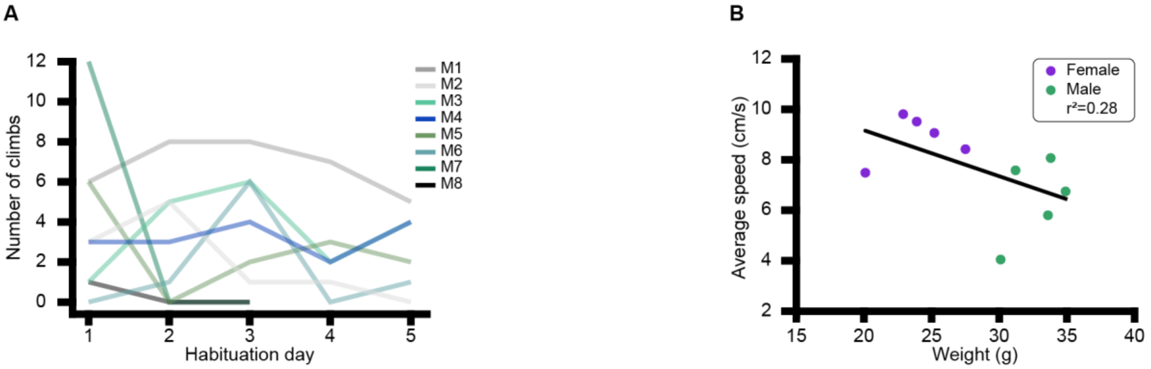

Figure S1. Habituation and weight. (A) Spontaneous climbs across habituation days for each mouse; only two mice did not spontaneously climb during habituation (not shown in figure). (B) Climbing speed as a function of weight for females (purple) and males (green), linear regression shows a negative trend indicating slower average speed was related to body weight.

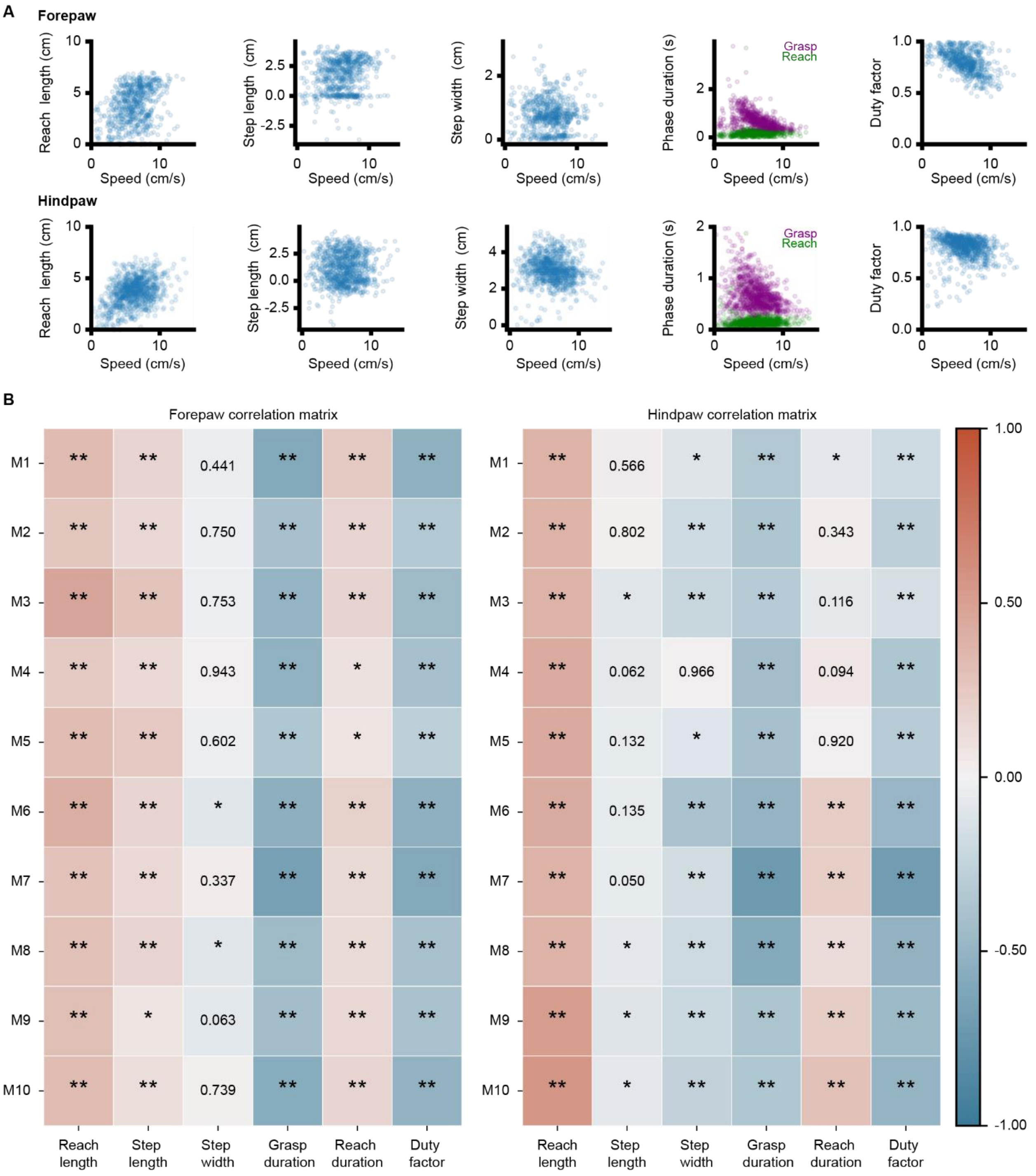

Figure S2. Correlations between speed and kinematic variables. (A) Example forepaw (top row) and hindpaw (bottom row) scatter plots showing relationship between speed and reach length, step length, step width, phase duration, and duty factor from one mouse. (B) Correlations for speed and kinematic variables for all mice (M1-M10) for forepaw (left) and hindpaw (right) data. Color coding of tiles represents correlation strength, while values represent significance of correlation (*p<0.5, **p<0.001).

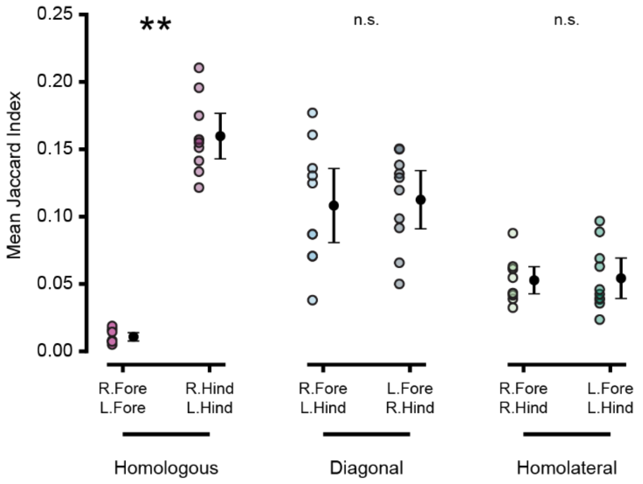

Figure S3. Temporal overlap of paw pairs on standard wall. Mean Jaccard index calculated across homologous (left) diagonal (centre) and homolateral (right) paw pairs for each mouse across all climbs. Data points to the left of the x-axis index indicate individual mouse values, with the group mean and 95% confidence intervals shown to the right of the x-axis index. (n=10 mice, n.s. p>0.05, **p<0.001, linear mixed-effects model with Bonferroni correction).

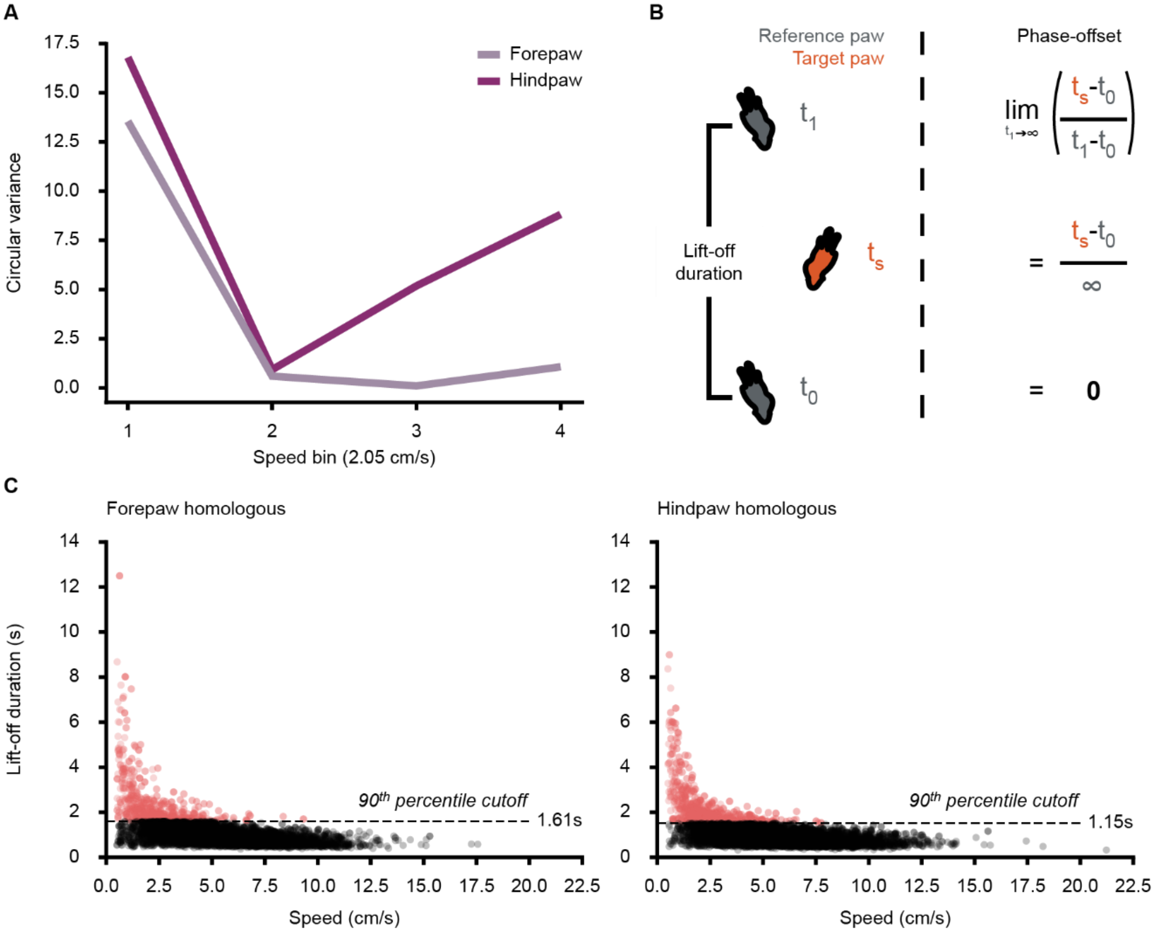

Figure S4. Lift-off duration increases at lower speeds. (A) Circular variance of homologous forepaw (light purple) and hindpaw (dark purple) pairs across speed bins (represents 2.05cm/s) showed greater variance in slowest bin. (B) Illustration of lift-off duration (right) for reference paw (grey) for phase-offset calculations with target paw (orange). As the lift-off duration increases (right), the phase-offset approaches 1: generating a false in-phase relationship. (C) Lift-off duration as a function of speed for phase-offset values between homologous forepaw pairs (left, n=7656 values from 10 mice), and hindpaw pairs (right, n=9278 values from 10 mice). Red points and black points indicate data points above and below the 90th percentile (dashed line), respectively.

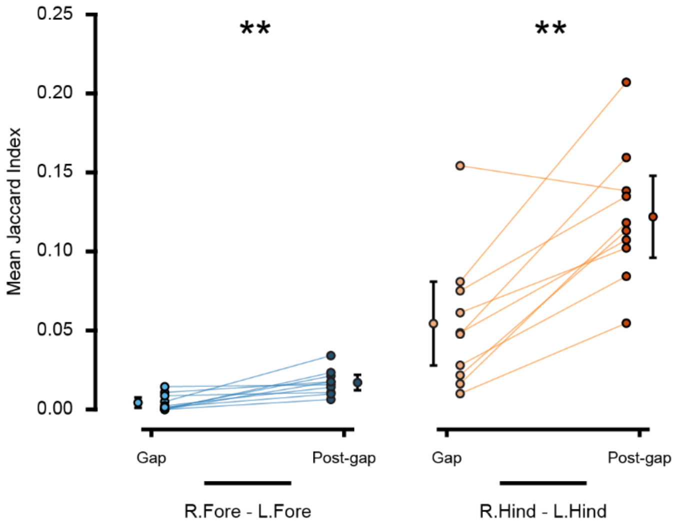

Figure S5. Change in temporal overlap of homologous pairs during gap cross. Mean Jaccard index calculated across homologous forepaw (left) and hindpaw (right) pairs for each mouse across all climbs. Data points between the x-axis indices indicate individual mouse values with shaded lines connecting individual data points between the two conditions (gap and post-gap). The respective group mean and 95% confidence interval are shown outside the corresponding x-axis index. (n=10 mice, **$p<0.001$, linear mixed-effects model).

Table S1. Network hyperparameters for pose estimation.

Parameter	Value
Model	
Max stride	32
Filters	16
Filters rate	2.00
Middle block	True
Up interpolate	True
Sigma	1.75
Output stride	2
Data	
Input scaling	0.35
Optimization	
Batch size	4
Epochs	200
Initial learning rate	0.0001
Minimum key hardpoints	2
Augmentation	
Rotation minimum angle	-90.00
Rotation maximum angle	90.00
Gaussian noise mean	5.00
Gaussian noise standard deviation	1.00
Contrast minimum gamma	0.50
Contrast maximum gamma	2.00
Brightness minimum value	0.00
Brightness maximum value	10.00
